# Robust Quantification of Polymerase Chain Reactions Using Global Fitting

**DOI:** 10.1371/journal.pone.0037640

**Published:** 2012-05-31

**Authors:** Ana C. Carr, Sean D. Moore

**Affiliations:** The Burnett School of Biomedical Sciences, College of Medicine, The University of Central Florida, Orlando, Florida, United States of America; Universidad Europea de Madrid, Spain

## Abstract

**Background:**

Quantitative polymerase chain reactions (qPCR) are used to monitor relative changes in very small amounts of DNA. One drawback to qPCR is reproducibility: measuring the same sample multiple times can yield data that is so noisy that important differences can be dismissed. Numerous analytical methods have been employed that can extract the relative template abundance between samples. However, each method is sensitive to baseline assignment and to the unique shape profiles of individual reactions, which gives rise to increased variance stemming from the analytical procedure itself.

**Principal Findings:**

We developed a simple mathematical model that accurately describes the entire PCR reaction profile using only two reaction variables that depict the maximum capacity of the reaction and feedback inhibition. This model allows quantification that is more accurate than existing methods and takes advantage of the brighter fluorescence signals from later cycles. Because the model describes the entire reaction, the influences of baseline adjustment errors, reaction efficiencies, template abundance, and signal loss per cycle could be formalized. We determined that the common cycle-threshold method of data analysis introduces unnecessary variance because of inappropriate baseline adjustments, a dynamic reaction efficiency, and also a reliance on data with a low signal-to-noise ratio.

**Significance:**

Using our model, fits to raw data can be used to determine template abundance with high precision, even when the data contains baseline and signal loss defects. This improvement reduces the time and cost associated with qPCR and should be applicable in a variety of academic, clinical, and biotechnological settings.

## Introduction

Since its inception, the polymerase chain reaction has markedly advanced molecular biology, perhaps more than any other single technique [1]–[3]. One common application of PCR is to amplify specific DNA targets of interest from complex mixtures so that a determination of the initial abundance can be made. Quantitative PCR is implemented by monitoring the increase in dsDNA product as a function of the number of thermal cycles and has evolved into a large industry that focuses on monitoring and analyzing product accumulation in real-time, usually with an increase in a fluorescent signal [4]. Commonly employed quantification methods include either fitting sigmoidal functions to the raw data or fitting linear functions to log-transformed data. The latter is considered more accurate because it displays less variance and gives reproducible estimates of the reaction efficiencies [5]–[12]. What is lacking in the field is a mathematical model that accurately predicts the accumulation of product throughout an entire reaction [13]. With a complete model, an entire qPCR data set can be used for template quantification and the influences of baseline adjustment and signal quality can be directly assessed by comparing real and synthetic data.

The polymerase chain reaction is, in theory, an exponential amplification of template DNA because during each thermal cycle a template becomes two more [2]. With this premise in mind, the accumulation of product can be modeled either exponentially (predicting raw data) or through a log transform, which linearizes exponential data [10], [11], [13], [14]. A sticking point during these analyses is that the true reaction efficiency, which is the efficiency of converting a template into two products during each cycle, remains elusive because much of the efficient amplification occurs before the observable data rises above background [12]. This problem can be partially alleviated by employing methods that report the accumulation of product at earlier cycles, before the reaction efficiency has substantially waned [15]. Unfortunately, increasing signal sensitivity with hyper-sensitive reporters comes at a substantial cost that frequently outweighs its advantages over less expensive methods.

Here, we present a simple model that accurately describes PCR throughout the entire reaction profile. Using this model, we were able to evaluate the influences of baseline adjustment errors, signal variations, and reaction efficiency and compare them to real experimental data. We demonstrate that using log-transforms of the data for quantification is invalid, despite the fact it is among the most accurate methods to date. Additionally, we show that a determination of target quantity can be accurately obtained by fitting a simulated model to the complete data set data without the need to extract an efficiency value, without the need for log transformation, and without concern for the profile shape or baseline value. This advancement also allows for quality checks of adjusted data that are based on an accurate description of the entire reaction, not just regions arbitrarily deemed important. The main impact of our approach is that fewer replicates are needed to obtain reliable estimates of template quantity. Thus, the cost and time associated with qPCR can be greatly reduced.

## Results

### A simple PCR model that describes the entire reaction

We derived a PCR equation that describes the product accumulation throughout an entire qPCR data set using three variable terms: the amount of template present after the previous cycle (*prev*), the maximum capacity of the reaction (*max*), and the apparent affinity of accumulated reaction inhibitors (*K_d_*) (**Information S1**). As with the mass action kinetic model that describes exponential PCR phases with two parameters [13], our model is recursive in that product accumulation is dependent on the amount of template present after the previous cycle (*prev*).
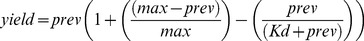
(6)


**Figure 1:**
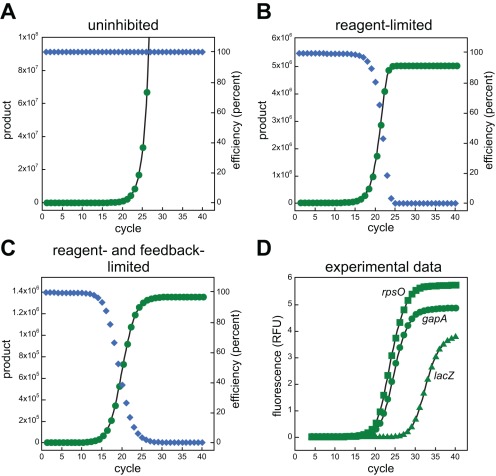
Comparing PCR equations. In panel **A**, product formation (green circles) is modeled to accumulate with a perfect, constant efficiency of 100% (blue diamonds) using equation 4 (**Information S1**). The simulated data was fit using non-linear regression using the same function (black line). Panel **B**, simulated data of a purely reagent-limited reaction is shown using equation 5 with a maximum product yield of 5×10^6^ (also fit to its function). Panel **C**, simulated data is shown using the PCR equation 6 with a *max* value of 5×10^6^ and a *K_d_* value of 5×10^5^. The efficiency terms at each cycle were extracted and plotted as blue diamonds. Panel **D** shows examples of real qPCR data fitted to equation 6 from amplifications using cDNA libraries generated from total *E. coli* RNA as templates. The resulting fitting values were: *rpsO*, *max*  = 25.148, *K_d_* = 1.6798, R^2^ = 0.99996; *gapA*, *max*  = 19.56, *K_d_* = 1.5753, R^2^ = 0.99998; *lacZ*, *max*  = 16.29, *K_d_* = 1.141, R^2^ = 0.99996.

The amplification efficiency (in parentheses) in each cycle varies. It changes from a value of two (100 % efficient) to a value of one (0 % efficient) as the PCR develops. Unlike other PCR models, this equation enables accurate modeling of entire data sets and is unaffected by cycle number, curve shape, or plateau height. Applying equation 6 to fit experimental data using nonlinear regression allows for determination of unique *max* and *K_d_* values for a wide variety of reactions (**Figure 1**).

### PCR is non-exponential and Log transforms of qPCR are non-linear

**Figure 2:**
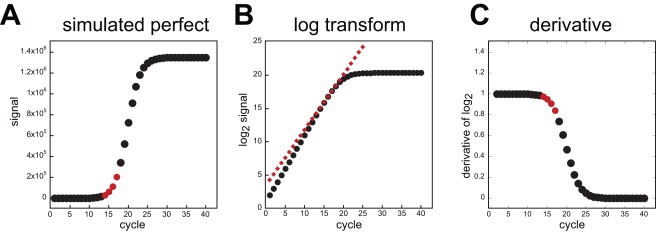
Simulated PCR and cycle threshold analysis. In panel **A**, PCR product formation was modeled according to equation 6 with max  = 5×10^6^ and *K_d_* = 5×10^5^. Four data points are highlighted that depict the region when the signal reached 1% of the final maximum observed. The data was transformed into log_2_ and the same 4 points were fit using linear regression. The slope and intercept from that fit were used to construct a straight line that was overlaid onto the log_2_ plot (panel **B**, diamonds). Note that the line does not predict the true progression of product at earlier cycles. Also, the earlier a reliable signal can be observed, the more accurate the estimation of the trend is. Panel **C**, the derivative of the log_2_ data. A value of 1 means that the efficiency was 100 % and the product doubled during that cycle. The region fitted for the cycle threshold analysis is marked in red and each value is lower than all preceding cycles.

Armed with an equation that accurately describes PCR, we were able to evaluate a very common method of qPCR analysis that relies on log-transformation of the data. In comparative “cycle threshold” analysis (C_t_), regions of log transforms of the data are fit to straight lines and the slopes and intercepts from these fits are then used to calculate reaction efficiencies and quantification cycles (C_q_). With the assumption that the reactions are purely exponential and that there is a constant efficiency, back-calculations are made from the differences in C_q_ that report the relative differences in starting abundance. We simulated perfect PCR data using equation 6 and evaluated it using cycle threshold analysis. The simulated data was transformed into log form and we analyzed the slopes and derivatives (**Figure 2**). Two points became abundantly clear: first, because the efficiency changed for each cycle, the log transforms are not truly linear, even though they visually appear so during early cycles. Second, once the product has accumulated to the point that the data leaves the apparent baseline, the reaction can be undergoing dramatic losses to its efficiency. Thus, calculating apparent reaction efficiency from data in this region always leads to an underestimation of the average efficiency in cycles preceding that window, a point that was previously predicted using sigmoidal analysis methods [16]. Moreover, using a straight line to fit threshold data points to estimate the starting amount is extremely sensitive to mis-adjusted baselines. This phenomenon has been also observed previously [10]. Below, we describe one major cause of such error and an appropriate correction. In summary, cycle threshold analysis suffers mainly from the fact that the efficiency always changes and that all of the calculations are based on a few data points near the baseline that have the weakest signal-to-noise ratio.

### Quantification of template abundance using regression

**Figure 3:**
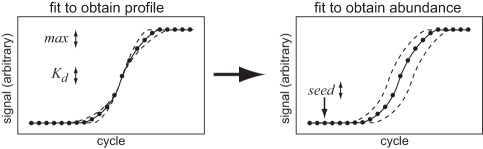
Two-step quantification. The PCR equation 6 is fitted to experimental data with weighting for stronger signals by floating the values *max* and *K_d_*. These values are then used to generate simulated data and a seed amount is computed that best superimposes the simulated data onto the experimental data. The relative values of seed correspond to the relative amounts of template DNA at that cycle.

To determine the relative amounts of template DNAs in a sample set, we employed an empirical calculation of template abundance in early cycles that allowed data modeled with the extracted *max* and *K_d_* terms to become superimposable with experimental data (detailed in Materials and Methods). To accurately determine *max* and *K_d_* for each reaction, experimental data was first fitted to equation 6 with fitting weight given to the brighter signals. These values were then used in a spreadsheet to model synthetic data using the same PCR equation. The differences between the modeled and experimental data for each observation was then calculated, squared, and summed. For the modeled data, the template amounts in an early cycle spreadsheet cell governed all subsequent values. Thus, by computationally searching for a template “seed” amount present after a cycle that minimized the differences between the modeled and experimental data, we obtained an accurate determination of the amount that was present in our real data at any point along the profile, even in the baseline region where the real signal was unobservable above background (**Figure 3**). In effect, by altering the amount of template present after an arbitrary early cycle, the position of the modeled curve was adjusted to fit on top of the experimental data. Once aligned, the template abundances in each cycle were available from the modeled spreadsheet data.

**Figure 4:**
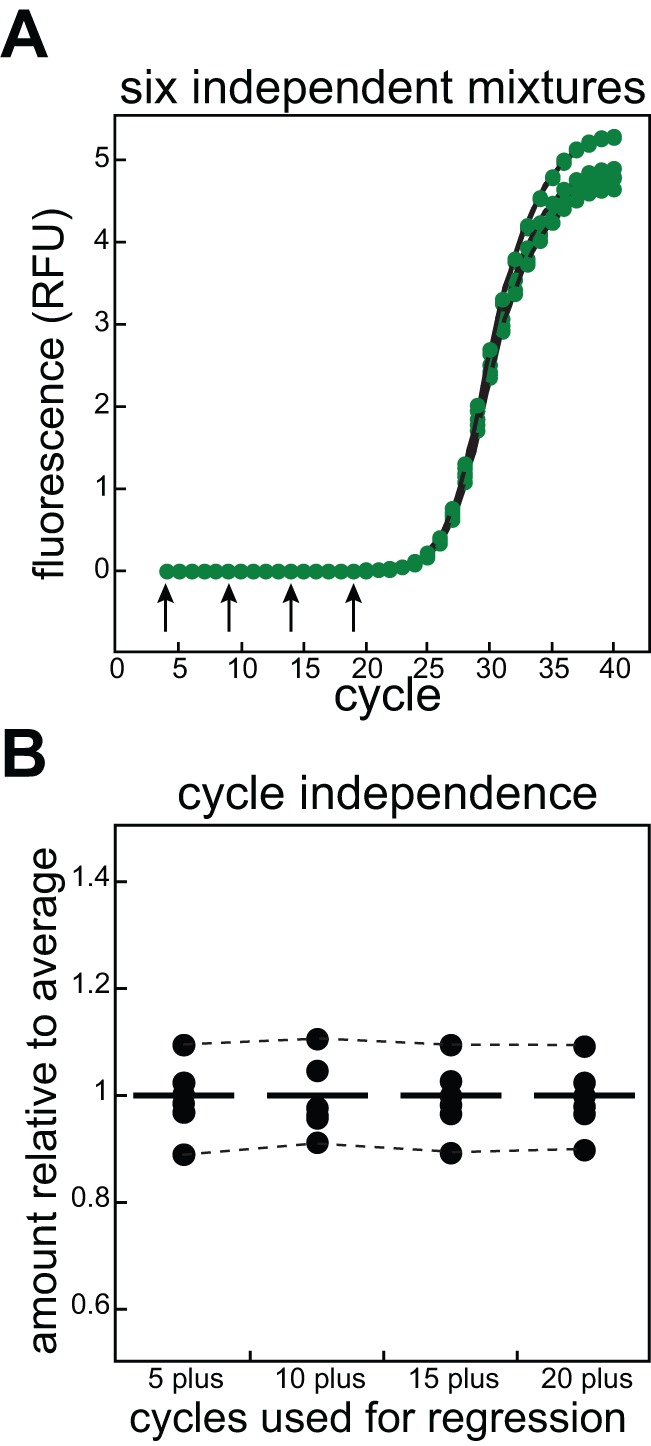
Using regression to determine relative abundance. Panel **A**, 6 independently-mixed qPCR samples that amplified cDNA from the *ompT* gene were fitted to PCR equation 6 to obtain *max* and *K_d_* values. These were then used in a spreadsheet to model synthetic data. The hypothetical DNA amount present as seeding doses in cycles 4, 9, 14, and 19 (arrows) were computationally floated to minimize the differences between the simulated and real data in cycles 5 plus through 20 plus respectively. The seed amounts present in cycles 4 and 19 differed by more than 3×10^4^. Panel B, The calculated seed amounts are plotted as fractions of the mean (straight lines) with dotted lines connecting the data from two outliers to highlight the small variance when different cycles were used in the regressions of the same sample.

The cycle selected for regression analysis does not significantly alter the resulting quantification because all reactions for a particular target scale fractionally in relation to their relative abundances with unique *max* and *K_d_* values governing the efficiencies in each PCR cycle. However, by selecting a cycle from the baseline region, before the detectable appearance of the product, a more intuitive relationship between data sets is obtained because the influence of *max* and *K_d_* is still minimal. To illustrate these points, we calculated relative abundance for a set of six independently-mixed qPCR reactions that amplified the same target from the same cDNA (**Figure 4**). Seed values in cycles 4, 9, 14, and 19 that gave rise to the best fit to the experimental data were then used to calculate abundance relative to the mean (**Figure 4B**). We did not include the first two data points in our calculations because they were observed to vary substantially from the baseline. Additionally, the starting material was not able to be exponentially amplified because only one strand of the target DNA was present in our cDNA mixtures and the first cycle or two would be needed to convert that DNA into suitable double-stranded templates.

We calculated a standard deviation from the average of 7.7% for the whole set of six reactions, which, considering the fact that these mixes were highly viscous and each sample was mixed independently, is quite small for qPCR analysis. Importantly, each individual reaction exhibited only small variations in the calculated amounts when different cycles were used for the regression analysis (for example, in **Figure 2B**, dotted lines connect the calculated amounts from the two outliers). The average standard deviation in each sample as a function of the cycle chosen for quantification was ∼0.9%, approximately the limit of our pipetting accuracy. Therefore, the seed cycle chosen for the quantification does not matter to any appreciable degree.

When we evaluated the ability of PCR equation 6 to fit a variety of experimental data, we observed that the values of *max* and *K_d_* were independent of the amount of baseline region that was included in the fitting procedure used to obtain them. Appreciable fitting error (R^2^<0.95) was only introduced when the entire baseline and approximately a third of the above-baseline amplification profile was omitted (not shown). Small baseline adjustment errors substantially affect conventional cycle-threshold analysis and can give rise to impossible efficiency terms (**Information S2, Figure S1**). Our analysis using global fitting is practically unaffected by baseline errors or signal loss (**Information S3, Figure S2**). Therefore, in principle, any arbitrarily chosen cycle in the baseline can be used to calculate abundance. Relative abundance can be determined between samples as long as the same cycle is chosen for seeding during each analysis.

### Quantification using global fitting is not affected by reaction efficiency or target abundance

Common methods to compare relative input abundance rely on an accurate estimation of reaction efficiency. In our model, the reaction efficiency changes during each cycle and it is not necessary to extract it because its influence becomes incorporated in the values of *max* and *K_d_*. To evaluate this notion, we computationally forced the efficiency to lower values by altering equation 6 such that it contained numbers less than one as the first term in the efficiency component (so the sum could not be 2 in any cycle). When the resulting equations were fit to real data, there were noticeable deviations in the fits and reductions in the R value were apparent when this term was 0.98 or less (fitting failed when the value dropped below 0.3, not shown). Each forced reduction in the efficiency term was met with changes to both *max* and *K_d_* in the resulting best fit, with dramatically increasing *K_d_* values when the term dropped below 0.9. Thus, the choice of one as the first term in the efficiency component of equation 6 is optimal for describing real data.

**Figure 5:**
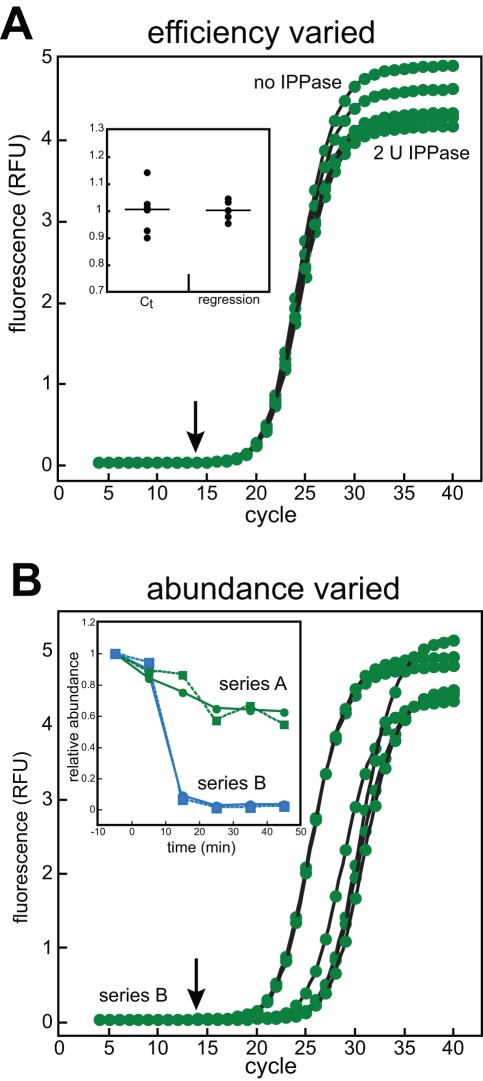
Regression analysis is insensitive to reaction efficiency and template abundance. Panel **A**, six qPCR mixtures targeting the *E. coli gapA* cDNA contained either 0, 0.01, 0.03, 0.12, 0.5, or 2 units of thermostable inorganic pyrophosphatase (New England Biolabs) added as 1 μL of a 20 μL reaction. The remaining volume was matched using the same storage buffer lacking enzyme. Fitting of the resulting amplification profiles with the PCR equation 6 (lines) yielded *max* and *K_d_* values that were used to calculate relative abundance in cycle 14 (arrow). The same data was also analyzed using the C_t_ method for comparison (inset). Panel **B**, a series of cDNA libraries were used as templates for qPCR that had been generated from an experiment in which the *gapA* mRNA levels changed drastically over time (series A and B). For clarity, only the data fitting curves are shown for series B in which the template abundance changed more than 20-fold. Both regression (circles) and C_t_ (squares) analyses were performed on the same data and the relative abundance plotted as a function of time (inset). Note that the resulting values described the same relative changes and trends, but that the regression method yielded smoother data.

As an additional test of the influence of reaction efficiency on quantification by our method, we deliberately altered PCR reaction efficiencies of the same target mixture. Literature reports of increased PCR yield when a thermostable inorganic pyrophosphatase (IPPase) was included in the reactions inspired us to test this enzyme in a qPCR series to see if we could drive the reaction forward by degrading the pyrophosphate, one of the two products of the chain reaction [17], [18]. Unexpectedly, the addition of IPPase reduced the apparent reaction yield (**Figure 5A**). This reduction in apparent yield was also observed when different targets were amplified (not shown). We do not know the cause of the reduction, but it is possible that this version of IPPase (purchased from a commercial source) either directly inhibited the reaction or the preparation contained an inhibitory ingredient that was not listed as a buffer component. Alternatively, the release of free phosphate could have impeded the reaction, lowered the binding affinity of the fluorescent reporter, or reduced the fluorescence efficiency. Nonetheless, the addition of the IPPase induced noticeable perturbations to apparent reaction efficiencies that were reflected as changes to both *max* and *K_d_*. Importantly, the resulting changes to the profile shapes did not appreciably influence the accuracy of the quantification by our regression method, but did reduce the accuracy of quantification using the common cycle-threshold (C_t_) method and mass action method (**Figure 5A** **, inset,** and not shown) [10], [13].

A final test of the analysis method was performed to assess the influence of target abundance on the resulting quantification. When serial dilutions of test samples are made (as is common for qPCR interrogations), all competing/influential factors are concomitantly diluted as well, which does not reflect an experimental situation. Real-world sample analysis rarely requires the 100,000-fold dynamic range that is accomplished by the typical application of 5 10-fold dilutions, which themselves amplify pipetting variance. Additionally, we showed earlier that the baseline length before the visible profile does not influence the calculation. Therefore, we sought to analyze data from real samples that had a cDNA amount changing while the rest of the cDNA library remained essentially constant.

During a previous investigation, we observed a dramatic decrease in the amount of mRNA encoding glyceraldehyde phosphate dehydrogenase in *E. coli* (encoded by *gapA*), in some cases to levels that were less than a twentieth of the normal amount present in a control. Because this change in message abundance was representative of what can be encountered in an analysis of transcript abundance, we analyzed a single, non-averaged qPCR data set of 12 reactions from 12 cDNA libraries and compared the resulting template abundances using either the C_t_ method or the global-fitting, regression method (**Figure 5B**). The output data are similar in scale, but the values from the cycle-threshold method are noisier in comparison the regression method. Also, unlike the regression method, the noise observed using the C_t_ method became more exaggerated in the comparison of samples that had large displacements in their amplification profiles. This phenomenon stems from the use of a power operation to determine relative abundances using C_q_ values of log-transformed data, which exponentially amplifies error.

In most cases, the regression method presented here should not change the conclusions stemming from other popular analysis methods, but it will reduce the scatter in data sets and reduce the number of required measurements. Overall, our successful modeling of a PCR reaction allows for the fitting of unmodified amplification profiles using two terms that represent processes having the most influence on reaction efficiency at each cycle. It is worth reiterating that this modeling revealed that PCR reactions do not stop solely from reagent depletion, which is a common assumption. This approach removes an enigmatic “black box” from qPCR analysis that should aid in teaching and training, it allows accurate quantification that takes advantage of all data in an amplification profile, and it is insensitive to errors in baseline assignment, dynamic signal quality, and reaction efficiency.

## Discussion

Noise in experimental data can be reduced by increasing the number of measurements because noise does not scale linearly with true signal. For example, to reduce random noise by half, the number of measurements needs to be squared [19]. Unfortunately, for investigators using qPCR to quantify DNA, this relationship means that if a two-fold reduction in error bars is required in a particular project, the number of measurements will need to increase from a typical number of 3 to 9 for each sample, thus squaring the cost and dedicated time as well. We describe a method that reduces the measurement noise so that differences between samples can be determined with fewer measurements.

Existing qPCR analysis methods can produce high data variance, which complicates the measurement of many targets from a large collection of cDNA libraries. We traced a major contributor of the variance to a contribution of improper automated baseline assignment and a very slight loss of fluorescence efficiency each time a measurement was made. In the raw data, the effect is nearly imperceptible, but in the log transforms used for the fitting during C_t_ analysis, the effect is dramatic and heavily distorts the early data points in the amplification profile. We mathematically calculated an appropriate correction and adjusted our data prior to C_t_ analysis, which reduced such variance (**Information S3**).

We also evaluated a useful software program from another group that automates the baseline adjustment to maximize the linearity of the log transformed data [10]. During those corrections, we noticed that the calculated efficiency terms were sometimes greater than 100%, which is impossible by our current understanding of PCR. We then questioned whether arbitrarily adding or subtracting values to experimental data because it created a desired linear trend in log-transformed data was appropriate. Without a model to accurately evaluate the influence of baseline adjustments, we had to rely on a decrease in variance between repeated samples as the only measure to show that we had taken steps in the right direction.

It was unexpected that a predictive behavior model has not been used previously for qPCR analysis that reflects the step-wise accumulation of product throughout the entire reaction. The various kinetic events that underlie the amplification step have been rigorously evaluated mathematically [5], [20]; however, such modeling fails to capture the increases in signals that arise from completed amplifications that are at equilibrium. Also, there are so many dynamic parameters in a complete kinetic analysis of PCR that fitting real data is intractable. A mass action exponential model was employed by others that predicts the data early in an amplification profile and yields an accuracy comparable to the C_t_ method [10], [13]. However, this method is similarly influenced by well-to-well variations in the profile shapes that stem from a collection of uncontrollable variables including optical precision, reaction volume, and a dynamic efficiency term.

Because PCR reaction profiles resemble sigmoids, several groups have developed various sigmoidal models in an attempt to extract efficiency and threshold values that can then be used for calculating relative abundance, despite the fact that there is no obvious sigmoidal process underlying the increase in signal [6], [9], [21]. As with any mathematical modeling, adding more variables to improve data fits is not necessarily warranted, and sigmoidal fitting methods are not as reproducible as log-transform threshold analysis when baselines are properly adjusted [10]. A fifth parameter in sigmoid analysis was implemented to account for asymmetry around the sigmoidal inflection point [9]. In our analyses, we see different inflection points in data for the same template in different wells of the same experiment, so the physical relationship between an infection point and the amount of template is not clear. We suspect the difficulties in fitting qPCR profiles with sigmoids arise because the transitions into and out of the dynamic region of the data are differentially influenced by the *max* and *K_d_* terms. The asymmetry around the inflection point indicated to us that there are at least two processes governing the cessation of a PCR reaction.

The implementation of reagent depletion as a modulator of efficiency made intuitive sense for a closed system. At first glance, one might expect that the *max* term should remain essentially constant between different samples when using the same master mix. However, this value is also influenced by the signal strength in each well, so differences in machine calibration, optical alignment, and reaction volumes can each influence the apparent yield in different measurements of the same target. It was the addition of the feedback-inhibition term that permitted highly accurate fitting. We are surprised that the entire mass action event could be described with a single “inhibitor” and a single apparent K_d_ value, especially considering that two dominant products, dsDNA and pyrophosphate, accumulate at different scales. For each mole of dsDNA produced in a typical qPCR experiment, there are approximately 200 moles of pyrophosphate liberated. Despite this, adding additional terms to the efficiency component of the equation did not improve the fitting accuracy to any degree that influenced the final quantification because experimental data is described very well with equation 6.

The lack of dependence on the length of the baseline is an important conclusion because it suggests that as long as a few baseline cycles are available for accurate global fitting, the timing of the appearance of the amplification profile (stemming from the abundance of the initial template) does not affect the calculations. Initial target abundance should only be a consideration in cases where there is a trace amount of target and competing side-reactions markedly influence the data. Therefore, comparisons of the melt-curves and product uniformity are still important to ensure that the correct dsDNA is being monitored and standard data quality guidelines should still be employed [14].

Remaining hurdles in accurate quantification now stem from true statistical variations in the amount of template added, from poorly-calibrated machines, and also from liquid handling. Commercial qPCR mixtures of enzyme, reporter, dNTPS, buffer, salts, and stabilizer substantially reduce sample-to-sample variation and allow reproducibility over long time scales. In our hands, accurately distributing the mixes containing primers to each sample well is challenging and variable because the mixtures are viscous and have high affinity for the plastic pipette tips and wells. This property also makes thorough pre-mixing of the input template difficult and so most mixing likely occurs during the first few cycles from thermal convection, which may also influence the measurement of apparent starting amount. Being appropriately trained in handling such liquids is crucial, and the importance of ensuring that consistent (rather than accurate) volumes are delivered to each well cannot be overemphasized. However, multiple measurements of the same sample can now have a greater impact on reducing scatter in abundance calculations because each individual determination can be made more accurately.

## Materials and Methods

### Quantitative PCR

Complementary DNA libraries were generated from *E. coli* total RNA using a commercial kit (Bio-Rad iScript cDNA synthesis kit). Commercial qPCR master mixtures were from various sources (Bio-Rad: IQ SYBR® Green Supermix or SsoFast EvaGreen® Supermix; Applied Biosystems SYBR Green® PCR master mix). Quantitative PCR was performed on several machines (Applied Biosystems 7500 Fast®, Bio-Rad iCycler®, Bio-Rad IQ®, and Bio-Rad MiniOpticon®). All reactions were run with 40 cycles and the target PCR products ranged from 90 to 120 base pairs.

### Data analysis

Cycle-threshold analysis was performed using either on-board software or exported and analyzed with or without additional baseline adjustments using the LinRegPCR software [10]. Sloping baseline adjustments and signal-loss-corrections were made using Microsoft Excel (**Information S2**). Global fitting to obtain *max* and *K_d_* was performed using Kaleidagraph (Synergy Software). The fitting was recursive (each ordinate value depended on the previous ordinate, not on the abscissa), so two adjacent columns of data were used, one containing the raw values from cycles 3 through 39, and the adjacent containing the data to be fitted with cycles 4 through 40. A final column contained the weights for each data point based on the relative intensities of the fluorescence. Kaleidagraph interprets a value of one as having the most weight and larger values having less weight. Therefore, weights were scaled linearly to match the relative brightness of each measurement compared to the maximum brightness observed in the reaction, which was usually the last data point. Weights were calculated using:
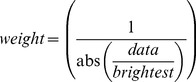
(1) Where the *weight* applied to a given data point was the absolute value (abs) of the current *data* point divided by the largest data point (*brightest*). Because we sought *max* and *K_d_* values that described the shape of the amplification profile as accurately as possible, weighting was implemented to lessen the impact of long or drifting baselines and weak signals. Fitting was accomplished by plotting the raw data *versus* the cycle number and activating non-linear regression using the PCR formula with weighting included. For each cycle, Kaleidagraph fitting required a table function to use a data column containing the template abundance from the previous cycle to calculate of the amount of product yield expected. Therefore, the following formula was used:
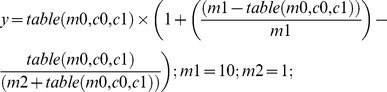
(2)Where *m0* is the cycle number, *m1* is *max*, and *m2* is *K_d_*. The data was present in columns *c0*, *c1*, *c2*, and *c3* contained the cycle number, the previous signal, the current signal, and the weights respectively. The plot was generated using columns *c0* and *c2*. The initial guesses for the non-linear fitting (10 and 1 in this case) were approximated to be on the same scale as the raw data.

The *max* and *K_d_* values from this weighted fit were then exported to an Excel spreadsheet. A “*seed*” cell contained an initial guess of the amount of signal that was present in the cycle immediately preceding the model window. A column of simulated data was then generated by having the first cell reference the seed cell and applying PCR equation 6 using the values of *max* and *K_d_* from the weighted fitting for that particular reaction. Each subsequent cell in the column used the same *max* and *K_d_*, but referred the amount present in the cell above it as *prev*. An example of the formula used for this progression is:
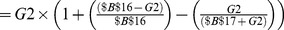
(3)Where *$B$16* was the cell containing *max*, *$B$17* was the cell containing *K_d_*, and *G2* was the cell above the current. When needed, subsequent columns of simulated data were generated that incorporated baseline drift or signal loss by referring to these “perfect” values. Real data was placed in a column and the difference between the simulated and real data was calculated and squared as an additional column. Finally, an output cell was created that contained the sum of the squared difference values. Using the included Solver GRG non-linear method in Excel, the value of the seed cell was drifted in order to minimize the sum-of-squares in the output column. When very small seed values were needed (for example when early cycles were being used for the quantification) both the convergence and constraint precision were adjusted to include more zeroes after the decimal. However, choosing a cycle near the beginning of the above-baseline signal did not require any adjustment for a solution to be found.

The Excel Solver reports the *seed* value, in arbitrary fluorescence units, that gave rise to the simulated data in the model being superimposed on the experimental data. These *seed* values were then used to calculate relative abundances between samples (schematized in **Figure 3**). In preliminary work, we evaluated floating all three terms (*seed*, *max,* and *K_d_*) simultaneously along with other terms that influence reaction efficiency and data quality. We concluded that using a weighted fit to obtain *max* and *K_d_* yielded terms that more accurately described the shape, and using non-weighted fitting for determining seed amounts yielded more reproducible data (not shown). Thus, we adhere to a two-stage fitting procedure.

## Supporting Information

Information S1 **Deriving a PCR equation.** A model for PCR product accumulation as a function of the maximum possible yield and the inhibitory influence of reaction products outlined.
(DOC)

Information S2 **Baseline adjustment.** The influence of incorrect baseline assignment on qPCR reaction data and the resulting quantification is detailed using simulated and real data for comparison.
(DOC)

Information S3 **Signal Loss.** An analysis of signal loss and its influence on both synthetic and real data is presented along with a derived correction for repeated, first-order decay.
(DOC)

Figure S1 **Baseline errors and their influence during data analysis.** Panel **A** shows the log_2_ transforms of simulated perfect qPCR data (circles) that were altered by adding either a small amount to each point (0.1 % of the maximum signal, “too high”, triangles) or that were raised above the baseline slightly and then lost signal every time a measurement was made (“too low”, squares). Note that the sample undergoing signal loss loses log transform data when the raw values become negative. Panel **B**, the derivative of the log data is plotted to illustrate that these small baseline errors dramatically influence the apparent reaction efficiencies. In panels **C** and **D**, experimental data is analyzed before and after a correction for signal loss. Unlike the uncorrected data, the log transform of the adjusted data exhibits a nearly-linear trend as the raw data leaves the baseline. Importantly, the derivative indicates that the apparent efficiencies of the corrected data trend towards the theoretical maximum, unlike the uncorrected data.
(EPS)

Figure S2 **Identifying and correcting signal loss.** Panel **A**, simulated data of a perfect reaction was modified such that 1% of the fluorescence signal was lost during each measurement (squares). The damaged data was then corrected using equation 8 (circles). Fits of the PCR equation 6 yielded *max* and *K_d_* values from the corrected data that were identical to those used to generate the the raw data (50 and 0.5 respectively). The *max* and *K_d_* values of the damaged data were each reduced (26.445 and 0.45519 respectively). The residuals of the fit to the damaged data are shown below. Panel **B**, experimental data before (circles) and after (triangles) manual correction for a linear sloping baseline. The inset shows the baseline region on a different scale to highlight the small signal loss in the raw data. The *max* and *K_d_* values for the uncorrected data were 25.419 and 1.2116 with an R^2^ of 0.99905. These values were 25.675, 1.2114, and 0.99918 for the corrected data. The residuals for the uncorrected (squares) and corrected data (circles) are displayed below. These residuals are typical of the fits to real data and indicate that either the model is incomplete or the raw data are not perfect despite attempted corrections.
(EPS)
