# Supplementary material for: Robust Quantification of Polymerase Chain Reactions Using Global Fitting
Source: PLoS One. 2012 May 31;7(5):e37640. doi: 10.1371/journal.pone.0037640 (PMC3365123; doi:10.1371/journal.pone.0037640)
Supplement: Information S1 — Deriving a PCR equation. A model for PCR product accumulation as a function of the maximum possible yield and the inhibitory influence of reaction products outlined. (DOC) [file pone.0037640.s001.doc]

INFORMATION S1

Development of a PCR equation that models all data

Most attempts to model PCR reactions begin with the consideration that the reaction is exponential in nature with a nearly-constant amplification efficiency in the cycles preceding the data that is used for quantitative analysis (typically near the region the signal begins to leave the baseline) [12]. Such approaches are based on the mathematical prediction that the reaction proceeds, in some form, following the equation:

**(4)** $yield=initial\times efficiency^{\#cycles}$

In this equation, the amount of starting material (initial) and the amplification efficiency govern the product yield after a certain number of thermal cycles [10]. In a perfect setting, the efficiency equals two, meaning that each template gives rise to two product dsDNAs per cycle. In practice, measured efficiencies are less than two and range typically from ~1.8-1.95 [10-12]. The fundamental problem with this simplified view of a PCR reaction is that the reaction is predicted to generate a purely exponential expansion and such a behavior is not observed in a real setting (Figure 1). Clearly, in a closed system, substrate reagents become limiting as the reaction proceeds. Therefore, we modified the basic PCR equation such that the efficiency-per-cycle was influenced by the amount of available reagents. Because the amount of remaining reagent is directly proportional to the amount of product that was formed in preceding cycles, the fractional remaining reagent at the beginning of any given cycle can be described by the operation:

$$\frac{\left(max-prev\right)}{max}$$

Where max is the maximum amount of product that could possibly form and prev is the amount of DNA product present after the previous thermal cycle. The PCR model was changed to predict only the yield of product formed in one cycle. The modified PCR equation then became:

(5) $yield=previous\times\left(1+\left(\frac{max-prev}{max}\right)\right)$

Here, the efficiency term in parentheses is dynamic and scales from a value near 2 (when very little product has been formed) to a value near 1 (no amplification, when all of a limiting reagent has been converted to product). When modeled, the resulting data from equation 5 yields the profile observed in Figure 1B. Notably, the reaction is nearly 100% efficient until product accumulates to the point that the reagent pool has been markedly influenced. The reaction displays a sharp transition from nearly-exponential product formation to a flat plateau because most of the reagent is consumed in the few cycles preceding max. Importantly, although this equation generates data that is reminiscent of qPCR data, it was unable to fit any of our experimental data.

Our investigation into the cause of this failure led us to reconsider the prevailing notion that PCR reactions stop because of reagent limitation. A common perception is that the oligonucleotide primers become limiting, most likely because their initial quantity was demonstrated to influence product yield in early reports of PCR [2,4]. When we measured the amount of dsDNA produced from PCR reactions that had plateaued and compared them to the available primer concentration, we observed that the product yields were only approximately 20-25% of the available primer and far below the amount of available deoxynucleoside triphosphates (not shown). Therefore, some other process must be responsible for stopping PCR reactions.

The law of mass action dictates that, in a closed system, an enzymatic reaction rate is necessarily reduced as product accumulates [S1]. This phenomenon is the reason why most kinetic analyses are performed from initial rates, before substantial product accumulates. Therefore, because PCR occurs in a closed system, we concluded that the efficiency of a PCR reaction must be also influenced by the amount of product that had been produced in previous cycles. In its simplest form, the product can be modeled as an inhibitor with an unknown affinity for the enzyme. It is established that the fractional occupancy of an inhibitor for an enzyme is described by [S2]:

$$occupancy=\frac{\left[inhibitor\right]_{\text{free}}}{\left(K_{\text{d}}+\left[inhibitor\right]_{\text{free}}\right)}$$

Where the unbound (free) concentration of inhibitor dictates the occupancy of the enzyme with respect to its equilibrium dissociation constant (K_d_). Conveniently, during PCR, the amount of such an inhibitor is also directly proportional to the amount of product formed in previous cycles. We then modified the model such that the per-cycle efficiency was influenced both by a limiting reagent as described in equation 5 and also by the accumulation of inhibitory product to arrive at the final PCR equation:

(6)

Now, two components govern the amplification efficiency at each cycle and each is solely dependent on the amount of product that was present at the end of the previous cycle. One effector changes from a value of one to zero and the other changes from a value of zero to one. Thus, the overall efficiency drifts from early values near 100% to near 0% as the product accumulates. Simulated data generated from equation 6 is shown in Figure 1C. Consistent with observed qPCR data and depending on K_d_, it displays a rounded transition from near-exponential amplification to a plateau that sits at ~25% of the maximum possible yield. An important point here is that after the first thermal cycle, the efficiency is never perfect, nor is it constant, which is consistent with previous calculations [6,16].

We tested this PCR equation for its ability to describe a variety of real experimental data using non-linear regression and floating only two variables, max and K_d_. The equation was able to very accurately describe every amplification profile we analyzed with R factors typically greater than 0.999 (Figure 1D). Accurate fitting was observed on data generated by older qPCR machines with dim lighting sources, on data that is scaled to ~5 (Bio-Rad) and data that is scaled to ~5 million (ABI), and on data generated with different fluorescence reporters. Interestingly, because we were using proprietary commercial qPCR master mixes, we had no ability to predict max; yet, the fitting returned max values that were approximately 4-fold higher than the observed plateaus, which was consistent with our simulated, perfect model in which the reaction ceased primarily from the accumulation of an inhibitory product. Additionally, the values obtained for K_d_ were approximately 1/10th of max. This outcome was also predicted from our modeling of a perfect reaction. Because the signal analyzed in qPCR is an arbitrarily-scaled fluorescence signal, the observed values of K_d_ have no direct physical link to the presence of any particular inhibitory product. Rather, they serve simply to control the slowing of the reactions as product accumulates. Each experimental reaction displayed a unique max and K_d_ that governed the shapes of the curves.

In preliminary attempts to find the amount prev in our model from any given observation, we rearranged equation 6 such that it was solved for prev and equated to all other values. However, solving for prev yields three solutions, two imaginary and one real. The real solution is exceedingly complex with 44 instances of exponentials. Therefore, very small errors in the data measurement become egregiously amplified during the solution.

**SUPPORTING REFERENCES**

S1 Moelwyn-Hughes EA, Pace J, Lewis WC (1930) The Kinetics of Enzyme Reactions: Schutz's Law. J Gen Physiol 13: 323-334.

S2 Mailman RB (2008) Toxicant-Receptor Interactions: Fundamental Principles. In: Smart RC, Hodgson E, editors. Molecular and Biochemical Toxicology. Hoboken: John Wiley & Sons, Inc. pp. 370-372.
