# Supplementary material for: Robust Quantification of Polymerase Chain Reactions Using Global Fitting
Source: PLoS One. 2012 May 31;7(5):e37640. doi: 10.1371/journal.pone.0037640 (PMC3365123; doi:10.1371/journal.pone.0037640)
Supplement: Information S2 — Baseline adjustment. The influence of incorrect baseline assignment on qPCR reaction data and the resulting quantification is detailed using simulated and real data for comparison. (DOC) [file pone.0037640.s002.doc]

INFORMATION S2

Baseline adjustment errors and their impact on data analysis

Viewing our qPCR data with the software on various machine models highlighted an obvious defect in some data sets. When the data was viewed in log form, early cycle data trended downward, then data disappeared, and the remaining data curved up from the gap into the region to be used for cycle-threshold analysis. Consecutively absent data from the log transform was caused by consecutively negative data. Because dsDNA was being produced in these early cycles, this trend was an impossibility. Another group had also noticed the influence of improper baseline adjustment and created a useful tool to automate the baseline assignment such that the earliest data above background was as linear as possible [10]. One feature of their approach is that the most linear pre-corrected data is used as a guide to adjust the data preceding it, to match the same linear trend as closely as possible. Satisfactorily, these adjustments reduced the variances in our analyses as reported by many groups. However, did we do the right thing? Was it justified to adjust the baseline to impart a linear trend using guide data from an adjacent region of the amplification profile? In effect, that method imposed an efficiency on preceding data. When we noticed that some output efficiency values were over 100%, we realized that relying on variance as the only guide to evaluate data correction could lead to bias.

We were inspired to develop the PCR equation 6 as a model to test the influence of incorrectly applied baseline adjustments, which appeared to be the root cause of the data defects. Once we had developed a model that accurately described real data, we added and subtracted baseline values to all data in the simulated, perfect set to observe the changes in the log transforms and, more importantly, their derivatives, which reported the apparent efficiencies (Figure S1). When a small baseline value of 0.1% was added to the simulated data, the log transforms of the data deviated from the perfect set and leveled off at early cycles (Figure S1A, “too high”). When the derivative of these data was plotted, the maximum apparent efficiency was well below the true efficiency in those same cycles (Figure S1B).

To recapitulate the experimental observation that the log data disappeared and reappeared, it was necessary to apply a non-uniform baseline adjustment to the data. When we added a fixed amount to all of the data and then subtracted a value from each data point that increased as a function of the number of cycles, we were able to mimic the curving disappearance/reappearance in the log-transformed data (Figure S1A). The downward curved trend in the log data that approached the linear region was visually indistinguishable from data that had been modified by removing a uniform amount from each point (not shown). Also, the apparent efficiencies in early cycles was above the theoretical limit (Figure S1B). Thus, downward or upward curve trends in the log transforms of regions where Cq is calculated are indicative of improper baseline assignments. The derivative curve shape is a reliable indicator of the quality of the data that can be used prior to employing a quantification method. The derivative data should trend to a level value that sits just under the theoretical maximum.

Unfortunately, with real data, low signal-to-noise in the earliest cycles causes the log and derivative plots to have scattered data. For the experimental data shown in Figure S1C and **S**1D, raw data was compared before and after a baseline adjustment that involved correcting both for a uniform loss (imposed by the machine automatically over-subtracting in an attempt to establish a zero baseline) and from a decline the in the signal strength that was a function of the number of cycles. The log-transform of the corrected data appears straight as it becomes detectable above background. Importantly, the derivative plot trends toward the theoretical limit in efficiency, unlike the uncorrected data (Figure S1D). Note that the data points with stronger signals (later in the reaction) are relatively unaffected by errors in baseline adjustment because the defect is small relative to the overall signal. The global fitting procedure described in the main body of the manuscript takes advantage of this feature and is practically unaffected by baseline errors. In fact, adding an artificial baseline to real data that was 20% of the maximum signal did not prevent a reliable analysis (not shown).
