# Supplementary material for: Robust Quantification of Polymerase Chain Reactions Using Global Fitting
Source: PLoS One. 2012 May 31;7(5):e37640. doi: 10.1371/journal.pone.0037640 (PMC3365123; doi:10.1371/journal.pone.0037640)
Supplement: Information S3 — Signal Loss. An analysis of signal loss and its influence on both synthetic and real data is presented along with a derived correction for repeated, first-order decay. (DOC) [file pone.0037640.s003.doc]

INFORMATION S3

Correcting for signal loss during cycling

In some cases, we observed experimental data from our and other groups that exhibit striking declines in the signals of the plateau regions as the cycling continued after the completion of the amplification stage. This observation should not be ignored, the product DNA is not depleting during those cycles, rather, the fluorescence reporter is most likely photobleaching. The bleaching occurs throughout the entire reaction each time a measurement is made and influences all of the data, not just the plateau region. It only becomes overt when the accumulation of product has slowed sufficiently (Figure S2A). In most cases, data that has been distorted by repetitive signal loss is indistinguishable from a normal qPCR profile (Figure S2B).

The loss in signal from photobleaching is a first-order process and, because measurements are made with repeated, nearly-consistent exposures to light, the amount of active fluorophore remaining after each measurement is a fixed fraction of what was present before the measurement [S3]. The cycle-dependent loss can be described by:

**(7)** $observed=real\times\left(signal remaining\right)^{cycle\#}$

In this scenario, the number of exposure cycles that have occurred prior to each measurement can substantially alter the data and influence reproducibility, especially when rarer templates require a greater number of cycles to be detectable. The steps to rearrange equation 7 to solve for real are:

1. divide by real: $\left(remaining\right)^{\#cycles}=\frac{observed}{real}$

2. take the log: $cycle\#\text{ }\times\log(remaining)=\log(observed)-\log(real)$

3. subtract log(observed): $cycle\#\text{ }\times\log(remaining)-\log(observed)=-\log(real)$

4. multiply by -1: $\log(observed)-cycle\# \times\log(remaining)=\log(real)$

5. choose a log base: $\log_{2}(observed)-cycle\#\text{ }\times\log_{2}(remaining)=\log_{2}(real)$

6. raising the equation to the selected log base (2 in this case) yields an equation that allows correction for the consistent signal loss:

(8) $real=2^{\left(\log_{2}\left(observed\right)-\left(cycle\#\text{ }\times\log_{2}\left(remaining\right)\right)\right)}$

Applying equation 8 to simulated PCR data that has undergone signal loss restores the normal appearance of the amplification profile (Figure S2A). The experimental challenge is to accurately determine the signal loss as a function of what was present before the measurement. Such a determination is difficult, and is made impossible if data has already been baseline adjusted. However, PCR equation 6 still fits such damaged data and extract values for max and K_d_ that allow for template quantification (Figure S2A). In a real setting, there is no clear indication that the data being analyzed has been distorted by such a dynamic process because the log and derivate plots barely change (Figure S2B, and not shown). Trended residuals of the fit to PCR equation 6 provide an indication that the data is non-ideal and this feature can be used to assess data quality.

Fluorescent reporters that are more stable are less prone to induce this artifact and that the commonly used SYBR^Ⓡ^ Green can noticeably bleach [S4]. Also, older machines with dimmer excitation lights spare the fluorophore at the expense of generating noisier data. These observations are the reason we implemented a weighting procedure to the data points with the highest signals during our fitting to obtain max and K_d_. As a precaution, the “loss per cycle” term from equation 7 can be added in the spreadsheet equation that generates the simulated data for the calculations of abundance and simultaneously floated along with the seed amount during the minimization of the sum of squares. Because the distorted data is still well-fit by equation 6, the solution should return a value very near 100% as the amount of active fluorophore remaining per cycle, even if that is known not to be the case.

These data defects have a high impact on C_t_ analysis accuracy, especially when the C_q_ between compared samples are separated by several cycles. Although it is beyond the scope of this report, it seems reasonable that an automated process could be implemented that applies the signal-loss-correction in conjunction with baseline assignments in an effort to minimize the residuals to the fit to equation 6. Another consideration is a loss in enzymatic activity per cycle, which was not explicitly included in our model. A loss in enzymatic activity is expected to be reflected as changes to the apparent *K_d_* of an inhibitor as a function of the number of cycles. We have not performed a thorough analysis of the trends in *K_d_* for the same target with widely-differing template abundances, which should reveal this effect.

**SUPPORTING REFERENCES**

S3 Axelrod D, Koppel DE, Schlessinger J, Elson E, Webb WW (1976) Mobility measurement by analysis of fluorescence photobleaching recovery kinetics. Biophys J 16: 1055-1069.

S4 Eischeid AC (2011) SYTO dyes and EvaGreen outperform SYBR Green in real-time PCR. BMC Res Notes 4: 263.
